# T203. ILLICIT DRUGS USE AND ULTRA-HIGH RISK (UHR) FOR PSYCHOSIS STATUS IN A LATIN-AMERICAN SAMPLE

**DOI:** 10.1093/schbul/sby016.479

**Published:** 2018-04-01

**Authors:** Mauricio Serpa, Alexandre Andrade Loch, Camille Chianca, Elder Freitas, Julio Cesar Andrade, Tania Maria Alves, Lucas Hortêncio, Martinus Theodorus van de Bilt, Wagner Gattaz, Wulf Rossler

**Affiliations:** University of Sao Paulo

## Abstract

**Background:**

In recent years, a number of investigations have evaluated the effect of cannabis use on the risk of presenting ultra-high risk for psychosis (UHR) status as well as its influences on transition rate, suggesting a dose-dependent interaction. On the other hand, the association between cocaine (snorted or smoked) - an increasing health issue in several countries worldwide - and the UHR state was not appropriately examined. Also, exposure to other psychotomimetic drugs, as amphetamines and lysergic acid diethylamide (LSD), has not been investigated yet. We sought to examine differences in the prevalence of drug use between UHR subjects and epidemiologic controls (EC).

**Methods:**

Over 2500 individuals from the city of São Paulo (Brazil), aged between 18 and 30 years old, were screened with the Prodromal Questionnaire. Subjects with scores higher than 18 points in the positive subscale were invited to be thoroughly assessed with the application of SIPS (Structured Interview for Psychosis-Risk Syndromes). Drug use (lifetime use, age of first use and more intense use) was assessed using South Westminster scale.

**Results:**

100 individuals presented UHR state; other 110 were enrolled as EC. A subsample of 50 UHR subjects and 82 HC with data on drugs consumption were evaluated herein. UHR subjects history of lifetime drug use was: 19 (38%) cannabis; 5 (10%) snorted cocaine; 1 (2%) crack; 1 (2%) amphetamine; 2 (6.9%) LSD. EC history of lifetime drug use was: 20 (24.4%) cannabis; 6 (7.3%) snorted cocaine; 0 crack; 2 (2.4%) amphetamine; 1 (1.2%) LSD. No differences were observed for snorted cocaine (p=0.589), crack (p=0.379), amphetamine (p=1.0), or LSD (P=0.167). At a trend level, cannabis lifetime use (p=0.096) was more prevalent in the UHR group. Additional analyses showed that UHR subjects initiate cannabis use at earlier age than EC (p=0.006). In this group, 20% of subjects had used cannabis prior to 15 years of age, in comparison to 3.6% in the EC group.

**Discussion:**

Our results reinforce the view that cannabis use is linked to psychosis risk and that subjects at early age of exposure are at greatest risk. Nonetheless, studies with larger number of participants are warranted to confirm our findings, particularly on the lack of association between less frequently consumed drugs and the UHR for psychosis state.

